# Umgang mit COVID-19 in der Notaufnahme: Erfahrungsbericht der interdisziplinären Notaufnahme des Universitätsklinikums Münster

**DOI:** 10.1007/s00063-020-00693-0

**Published:** 2020-04-22

**Authors:** D. O. Wennmann, C. P. Dlugos, A. Hofschröer, M. Hennies, J. Kühn, W. Hafezi, S. Kampmeier, A. Mellmann, S. Triphaus, J. Sackarnd, P. Tepasse, M. Keller, H. Van Aken, H. Pavenstädt, P. Kümpers

**Affiliations:** 1 Medizinische Klinik D, Allg. Innere Medizin und Notaufnahme sowie Nieren- und Hochdruckkrankheiten und Rheumatologie, Universitätsklinikum Münster, Münster, Deutschland; 2 Institut für Virologie, Universitätsklinikum Münster, Münster, Deutschland; 3 Institut für Hygiene, Universitätsklinikum Münster, Münster, Deutschland; 4 UKM Infrastrukturmanagement, Bereich Projetentwicklung und Projektmanagement, Universitätsklinikum Münster, Münster, Deutschland; 5 Klinik für Kardiologie I: Koronare Herzkrankheit, Herzinsuffizienz und Angiologie, Universitätsklinikum Münster, Münster, Deutschland; 6 Medizinische Klinik B für Gastroenterologie und Hepatologie, Universitätsklinikum Münster, Münster, Deutschland; 7 Klinik für Anästhesiologie, operative Intensivmedizin und Schmerztherapie, Universitätsklinikum Münster, Münster, Deutschland; 8 Universitätsklinikum Münster, Münster, Deutschland

**Keywords:** COVID-19, Notaufnahme, Sars-CoV‑2, Rachen-Abstrich, Patientenlenkung, COVID-19, Emergency room, Sars-CoV‑2, Throat-swab, Patient allocation

## Abstract

Mit der COVID-19-Pandemie stehen die Notaufnahmen als Schnittstelle der ambulanten und stationären Krankenversorgung vor einer großen Herausforderung. Die Dynamik der Pandemie zwang die Notfallversorgung des Universitätsklinikums Münster zu umfassenden Anpassungsprozessen, die in kürzester Zeit erfolgen mussten. Dazu gehörte die Etablierung einer ambulanten Coronateststelle und einer studentischen Telefonhotline. Innerklinisch wurden neue Isolationskapazitäten in der Notaufnahme sowie eine eigene COVID-19-Station eingerichtet. Der Patientenfluss wurde durch Flussdiagramme sowohl für den ambulanten als auch für den stationären Bereich neu geregelt. Das allgemeine und spezielle Notfallmanagement wurde für die reibungslose Versorgung COVID-19-positiver Patienten optimiert und das Personal in der Benutzung von Schutzausrüstung trainiert. Dieser Erlebnisbericht soll anderen Notaufnahmen in der Vorbereitung auf die COVID-19-Pandemie unterstützen.

## Abstract (en)

With the COVID-19 pandemic, emergency rooms are faced with major challenges because they act as the interface between outpatient and inpatient care. The dynamics of the pandemic forced emergency care at the University Hospital Münster to extensively adjust their processes, which had to be carried out in the shortest time possible. This included the establishment of an outpatient coronavirus test center and a medical student-operated telephone hotline. Inside the hospital, new isolation capacities in the emergency room and a dedicated COVID-19 ward were set up. The patient flow was reorganized using flow diagrams for both the outpatient and inpatient areas. The general and special emergency management was optimized for the efficient treatment of COVID-19-positive patients and the staff were trained in the use of protective equipment. This report of our experience is intended to support other emergency departments in their preparation for the COVID-19 pandemic.

## Einleitung

Das Bundesland Nordrhein-Westfalen (NRW) ist von der COVID-19-Pandemie schwer betroffen. Hier kam es im Kreis Heinsberg nach den dortigen Karnevalsfeiern zum größten Ausbruch im Bundesgebiet. In der Folgezeit wurde NRW ein Hotspot für COVID-19 und war bis vor wenigen Tagen das Bundesland mit den meisten gemeldeten Fällen [1]. Die COVID-19-Pandemie stellt insbesondere die innerklinische Akut- und Notfallmedizin vor neue, bisher ungekannte Herausforderungen. Die Dynamik der Entwicklung und die Anpassung der Strukturen in der Patientenversorgung sind für Notaufnahmen und Intensivstationen weltweit eine neue Erfahrung [2]. Im Folgenden möchten wir chronologisch über unsere bisherigen Erfahrungen mit dem neuen Coronavirus SARS-CoV‑2 („severe acute respiratory syndrome coronavirus 2“) und über die daraus resultierenden Maßnahmen der interdisziplinären Notaufnahme des Universitätsklinikums Münster (UKM) berichten.

Die Notaufnahme wurde erstmalig Ende Januar mit dem Thema SARS-CoV‑2 konfrontiert. Am 23.01.2020 meldete sich eine Patientin nach Rückkehr von einer Dienstreise nach China telefonisch beim diensthabenden Internisten und schilderte Husten, Abgeschlagenheit und Gliederschmerzen – letztlich war SARS-CoV‑2 jedoch nicht nachweisbar. Am Folgetag finalisierten wir eine erste Verfahrensanweisung zum Transport von Blutproben, Hygienemaßnahmen und Isolationsmöglichkeiten von fraglich infizierten Reiserückkehrern. Die erste Probe eines Rachenabstrichs wurde am 31.01.2020 in die Virologie der Charité verschickt. Zeitgleich wurde im Notaufnahmeteam ein erstes Flussschema zum Umgang mit SARS-CoV-2-Verdachtsfällen kommuniziert. Ferner etablierten wir einen UKM-internen E‑Mail-Verteiler zu SARS-CoV‑2, um einen reibungslosen Informationsfluss zwischen den Fachdisziplinen Notfallaufnahme, Virologie, Hygiene und Mikrobiologie zu gewährleisten und insbesondere die Meldung von klinischen Verdachtsfällen als auch labordiagnostisch bestätigter Fälle beim Gesundheitsamt sicherzustellen. Vermerkt wurden Name des Patienten, Symptome, Kontaktdaten und die Anamnese, die den Abstrich begründete.

Ab dem 01.02.2020 war dann eine PCR-Testung auf SARS-CoV‑2 in der Virologie des UKM verfügbar, nachdem Positivkontrollen aus der Charité zur Etablierung beigebracht wurden. Verdachtsfälle (zu diesem Zeitpunkt ausschließlich China-Rückkehrer [3]) wurden zunächst ab dem 03.02.2020 in der RTW-Halle der Notaufnahme durch das geöffnete Autofenster ersteingeschätzt, abgestrichen und – wenn vertretbar – unmittelbar in die häusliche Quarantäne geschickt. Diese Praxis war für ca. 3 Wochen bei Reiserückkehrern aus China machbar und praktikabel. Mit steigenden Fallzahlen von COVID-19-Patienten in Norditalien [4, 5] nach dem 21.02. und dem beginnenden COVID-19-Ausbruch im Kreis Heinsberg (NRW) kam es zu einem deutlichen Anstieg der Patientenvorstellungen zur Abklärung einer möglichen SARS-CoV-2-Infektion. Parallel dazu erschwerten unzählige Anrufe besorgter Bürger auf dem Telefon des diensthabenden Internisten die Patientenversorgung erheblich. Die Dynamik der Ereignisse führte zur Einrichtung eines ab dem 27.02.2020 täglich tagenden Krisenstabs, in den sich die Notaufnahme von Anfang an sehr aktiv eingebracht hat.

## Etablierung einer Coronateststelle und -hotline

**Figure 1:**
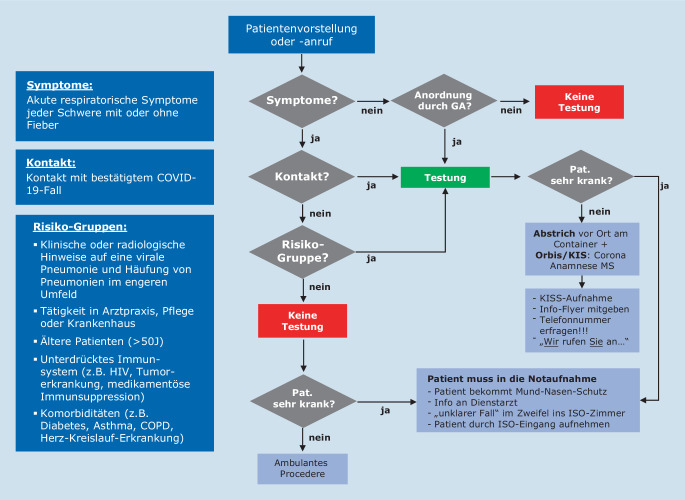

**Figure 2:**
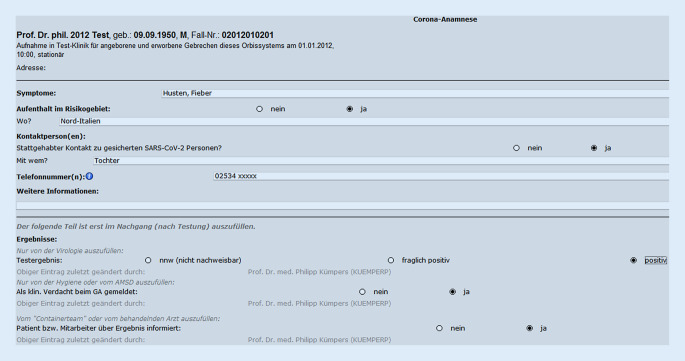

In Folge der Schließungen mehrerer Kindertagesstätten und Schuleinrichtungen in Münster kam es am 27.02.2020 erneut zu einem drastischen Anstieg von Telefonanrufen. Hierbei standen allgemeine Fragen zu SARS-CoV‑2, Verhaltensweisen, aber auch Fragen zur Diagnostik im Vordergrund. Die normalen Arbeitsabläufe innerhalb der Notaufnahme waren deutlich beeinträchtigt. Um wieder eine Fokussierung auf die Kernfunktionen einer Notaufnahme zu ermöglichen, wurden binnen 24 h folgende Prozesse etabliert: In räumlicher Nähe zur Notaufnahme, allerdings außerhalb des Klinikums, wurden 2 Bürocontainer aufgestellt, in denen wir unsere erste Coronateststelle einrichteten. So konnte bereits am 28.02.2020 ein Team aus je einer Pflegekraft, einer medizinischen Fachangestellten (MFA) sowie einem Arzt pro Schicht, im 2‑Schicht-Modell, von 08.00–20.00 Uhr an 7 Tagen in der Woche seine Arbeit aufnehmen. Initial erfolgte die Besetzung durch Internisten des Klinikums. Im Verlauf konnten nach Einführung von Videotutorials sämtliche klinischen Fachdisziplinen für den sog. „Coronacontainerdienst“ gewonnen werden. Zum Schutz des Personals erfolgt die Kommunikation mit möglichen Infizierten über eine Gegensprechanlage bei geschlossenem Fenster. Der Arzt erhebt hierbei die Anamnese. Diese orientiert sich an den gültigen Empfehlungen des Robert Koch-Instituts (RKI) in Form eines adaptierten Flussschemas (Abb. 1), das täglich aktualisiert und über das Intranet der Klinik zur Verfügung gestellt wird. Zur Dokumentation wurde im Krankenhausinformationssystem (KIS; in unseren Fall AGFA Orbis, Agfa HealthCare GmbH, Bonn, Deutschland) das „Corona-Anamnese-MS“(CAMS‑)Formular etabliert. Dieses dient als zentrales Kommunikationsinstrument zwischen der Virologie, der Hygiene und dem ärztlichen Personal der Testungsstelle. Zudem ermöglicht es die Dokumentation der telefonischen Befundmitteilung und dient schließlich als Kurzarztbrief (Abb. 2). Sofern die Indikation eines Abstrichs gegeben war, erfolgte die administrative Aufnahme durch die MFA über ein außerhalb des Containers installiertes Kartenlesegerät. Der Abstrich wurde außerhalb des Containers durch eine Pflegekraft in entsprechender Schutzkleidung (Schutzkittel, Einmalhandschuhe, FFP2-Maske und Schutzbrille) durchgeführt. Im Schnitt wurden so zwischen 80 und 130 Abstriche pro Tage durchgeführt. Durch das Containerteam erfolgte eine zeitnahe telefonische Befundmitteilung. Aufgrund der sehr frühzeitig verfügbaren Testung konnten wir glücklicherweise ein Welle von 35 positiven Ischgl-Rückreisenden abfangen, die alle einen Super-spreader*-*Phänotyp zeigten [6].

**Figure 3:**
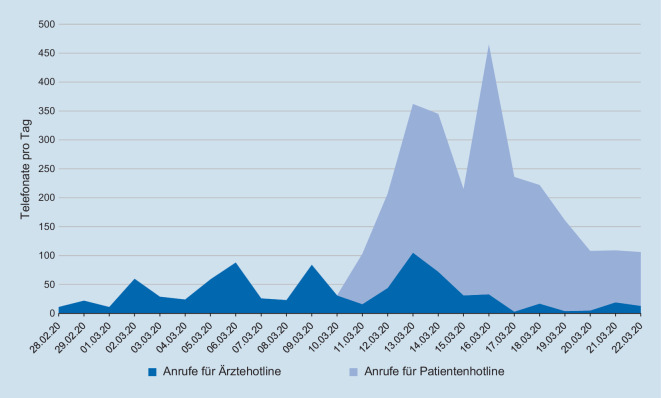

Aufgrund der Vielzahl der Anrufe im Coronacontainer konnte nach einem Aufruf durch den Studiendekan der Medizinischen Fakultät Münster kurzfristig eine studentische Telefonhotline aufgebaut werden. Nach zügiger Einarbeitung, täglich aktualisierten Flussschemata sowie engmaschiger ärztlicher Betreuung standen den Anrufern vorübergehend bis zu 3 Medizinstudierende gleichzeitig (2-Schicht-System) in der Zeit von 08.00–20.00 Uhr an 7 Tagen in der Woche kompetent zur Verfügung (Abb. 3).

**Figure 4:**
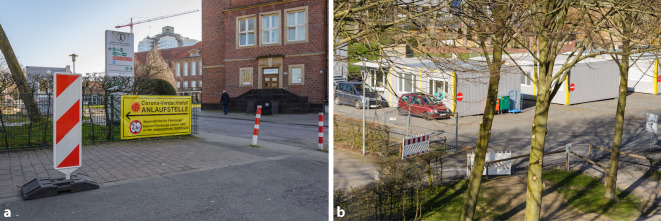

In Erwartung einer weiteren Zunahme von Verdachtsfällen erfolgte am 15.03. der Entschluss zur Etablierung eines 3‑spurigen Drive-in-Container-Parks mit deutlich besserer Zuwegung (Abb. 4a, b). Bereits am 20.03. konnten hier die ersten Abstriche durchgeführt werden. So können mögliche Infizierte in ihren Autos verbleiben und das Infektionsrisiko Dritter weiter minimiert werden. Die Sondierung der Abstrichindikation, Terminierung und Einweisung in das Drive-in-Konzept erfolgt durch die Studenten der Hotline.

Mit Etablierung der SARS-CoV-2-PCR wurde fortwährend die Logistik des Probentransfers zum (dezentral gelegenen) Institut für Virologie optimiert. Eine 2‑stündliche Abholung und zahlreiche kleine Verbesserungen („fast track“ für eilige Proben) ermöglichen derzeit eine realistische „turnaround time“ von ca. 4–6 h, je nach Tageszeit. Ebenfalls durch das Containerteam erfolgt die telefonische Benachrichtigung der Patienten spätestens am Folgetag, auch am Wochenende.

## Diagnostik und Allokation von Verdachtsfällen

Durch die neu geschaffenen Anlaufstellen für fraglich SARS-CoV-2-Infizierte wurde die Notfallaufnahme deutlich entlastet. Die Bürger verhielten sich erstaunlich diszipliniert und beschäftigten die Notaufnahme nicht mit Krankheitsbildern, die fachärztlich ambulant betreut werden können. Dennoch wurden nicht wenige Patienten vom Containerteam aufgrund festgelegter Kriterien zur Akutdiagnostik in die Notaufnahme weitergeleitet. Zudem häuften sich Patienten mit Atemweginfekten, die entweder rettungsdienstlich oder hausärztlich eingewiesen wurden. Da die Kapazität der vorhandenen Isolationszimmer mit Druckumkehr dafür nicht ausreichte, wurden zunächst 3 reguläre Untersuchungsräume, im Verlauf dann 2 weitere Observationszimmer auf Unterdruck umgestellt. Der Notaufnahme standen somit kurzfristig 10 Isolationsbetten zur Verfügung, von denen sich 5 in einem einigermaßen abgetrennten Isolationsbereich befinden. Hier können bisher nahezu alle höhergradigen Verdachtsfälle isoliert werden. Um Verdachtsfälle im KIS, wie dies bereits für MRSA-Besiedlung etc. üblich ist, hervorzuheben, führten wir eine farbliche Kennung ein. Fraglich infizierte Patienten konnten so in der Notaufnahme erstversorgt und eine entsprechende Diagnostik eingeleitet werden. Nachdem uns anfänglich noch ein positiver Alternativbefund (z. B. Influenza A, humanes Metapneumovirus oder Respiratory Syncytial Virus) in der Point-of-Care-PCR-Diagnostik (FilmArray® Respiratory Panel, BioMerieux, Nürtingen, Deutschland) zum Verzicht auf eine SARS-CoV-2-Diagnostik ermutigte, erschien uns diese Praxis mit steigenden Zahlen positiver Ergebnisse im Coronacontainer sehr bald zu unsicher. Die vom RKI publizierten Kriterien zur Testung bei begründeten Verdachtsfällen (ehemals Kriterium 1 und 2) waren für den regulären Betrieb in der Notaufnahme jedoch nicht hinreichend praktikabel, da auch Patienten ohne Reiseanamnese oder Kontakt zu einem COVID-19-Fall von den Fachabteilungen nicht ohne SARS-CoV-2-Testung auf die Stationen übernommen wurden. Daher wurden bald alle Patienten mit Fieber oder respiratorischen Symptomen vor Hospitalisierung auf SARS-CoV‑2 getestet und bis zum Eintreffen eines negativen Ergebnisses (formal: nicht nachweisbar, nnw) isoliert. Zum Screening implementierten wir einen Fragebogen, der häufige Symptome (Fieber, Husten, Dyspnoe, Myalgien, Halsschmerzen, Diarrhö, Geruchs- und Geschmacksveränderungen), Reiseanamnese und COVID-19-Kontakte abfragt und verpflichtend von den Triagemitarbeitern ausgefüllt wird. Sobald eines der Kriterien positiv beantwortet wird, erfolgen umgehend eine Schutzisolation und eine PCR-Testung.

Für Verdachtsfälle liegen spezielle COVID-Aufnahme-Pakete (EKG-Elektroden, Labor-Monovetten, periphere Verweilkanüle, Rachenabstrich, Blutkultuflaschen etc.) bereit, damit die Aufnahme möglichst zeitnah en bloc und dementsprechend ressourcenschonend durchgeführt werden kann. Zur Abschätzung des Krankheitsverlaufs wurde im Labor-Order-Entry-Modul für die Notaufnahme ein Laborstandard „COVID-19 Evaluation“ mit Differenzialblutbild, Ferritin, Interleukin‑6, Procalcitonin, LDH, D‑Dimeren und hochsensitivem Troponin hinterlegt. Auch wenn eine multinationale Validierung dieser Parameter noch aussteht, wird dieses Aufnahmelabor bei allen SARS-CoV-2-positiven Patienten sowie bei allen harten Verdachtsfällen abgenommen [7–9].

Zur Vermeidung unnötiger Verzögerung von Diagnostik und Therapie wurden ab dem 16.03. mit allen Funktionsabteilungen intensive Gespräche bezüglich der Gewährleistung einer schnellen und dennoch für alle Beteiligten sicheren Versorgung von COVID-19-(Verdachts‑)Patienten geführt: u. a. Endoskopie, Herzkatheterlabor, Radiologie, OP und Kreißsaal. Neben der Erstellung spezifischer SOP wurden spezielle COVID-19-Telefonnummern in den entsprechenden Abteilungen abgesprochen und transparent an alle Beteiligten kommuniziert. Dies gewährleistet eine unmittelbare Erreichbarkeit der COVID-19-Verantwortlichen der jeweiligen Abteilung. Alle Fachabteilungen optimierten zudem interne Abläufe, identifizierten geeignete Räumlichkeiten und schulten Hygienemaßnahmen in enger Rücksprache mit dem Institut für Hygiene. In den entsprechenden Anforderungsformularen des Krankenhausinformationssystems wurde bei der Abfrage der „Infektiosität“ eine eigene „checkbox“ für V. a. COVID-19 ergänzt.

**Figure 5:**
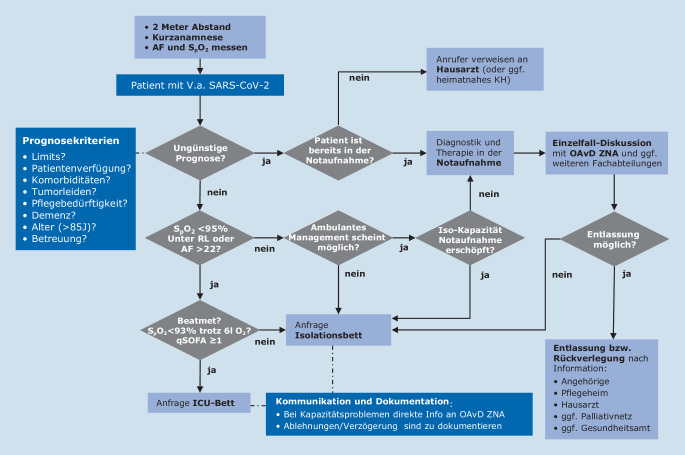

Die anfänglich lange Dauer bis zum Erhalt der PCR-Ergebnisse führte schnell zu einer Stagnation des Patientenabstroms (engl.: „exit block“), da jeder Patient mit Fieber und/oder respiratorischen Symptomen bis zum negativen Befund auf der Notaufnahme in Isolation verblieb. Geprägt von den Berichten der italienischen Kollegen wurde ab dem 06.03. im Krisenstab des UKM die Einrichtung einer COVID-19-Station vorbereitet. Aufgrund hygienischer und organisatorischer Aspekte wurde hierfür die Komfortstation des UKM ausgewählt. Innerhalb weniger Tage erfolgte eine bauliche Trennung der Station, die Klimaanlage wurde aufgerüstet und ein Ärzteteam, bestehend aus Anästhesisten und Internisten, wurde für einen Schichtdienst (2 OA+ 2-2-2) abgeordnet. Mitarbeiter der Hygiene begleiteten den Umbau und trainierten die Anwendung der persönlichen Schutzausrüstung mit den Mitarbeitern. Somit stand ab dem 18.03.2020 eine Station mit 18 Monitorbettplätzen zur Verfügung. Bei Bedarf kann die Station modular auf bis zu 64 Betten erweitert werden. Zusätzlich stehen 15 Isolationsbetten auf der Infektionsstation des Universitätsklinikums zur Verfügung. Für schwerkranke COVID-19-Patienten wurde die internistische Intensivstation gesplittet und der Teil mit Schleusenzimmern vorsorglich freigehalten. Anhand eines Allokationsalgorithmus (Abb. 5) können COVID-19-(Verdachts‑)Patienten zügig und für alle nachvollziehbar von der Notaufnahme auf die Infekt- oder Intensivstationen weiterverlegt werden, sodass die Handlungsfähigkeit der Notaufnahme weitestgehend gewährleitet ist.

Zusammenfassend verfahren wir zurzeit mit Verdachtsfällen innerhalb der Notaufnahme wie folgt: Klinisch stabile Patienten werden nach Anamnese, Untersuchung, Blutentnahme und Einsendung eines Abstrichs auf SARS-CoV‑2 nach Hause entlassen (Ergebnis wird telefonisch nachberichtet). Verdachtsfälle oder bestätigte Fälle, die eine medizinische Aufnahmeindikation erfüllen, werden auf die Isolationsstationen verlegt, dort entsprechend medizinisch versorgt und bei negativem Ergebnis entisoliert und verlegt. Kritisch kranke Patienten werden auf die Intensivstationen verlegt (Abb. 5). Bislang geht dieses Konzept in der Praxis auf, wobei insbesondere die Verfügbarkeit des sog. Clean-Teams für die Scheuer-Wisch-Desinfektion der Untersuchungs- und Patientenzimmer in der Notaufnahme frühzeitig erhöht werden musste.

Unabhängig von der räumlichen Allokation der (Verdachts‑)Fälle wird derzeit zunehmend die sehr frühzeitige thorakale Computertomographie zur Differenzialdiagnostik diskutiert. Gerade bei schlechter Verfügbarkeit bzw. langer „turnaround time“ der PCR-Diagnostik hilft die CT-Diagnostik zur frühzeitigen Diagnosestellung einer viralen Pneumonie. Bei aktuell sehr schnell verfügbarem PCR-Ergebnis (4–6 h) führen wir bei uns derzeit CT nur bei negativem PCR-Ergebnis und weiterhin bestehendem Verdacht auf COVID-19 durch – die Patienten liegen zu diesem Zeitpunkt meist schon auf den Infektionsstationen. Um zusätzliche CT-Kapazitäten zu schaffen, wurden unter anderem Untersuchungszeiten an Geräten reserviert, die normalerweise nicht in die Notfallversorgung integriert sind (z. B. SPECT-CT und PET-CT).

Andersherum werden alle Notfallpatienten mit radiologischen Zeichen einer Pneumonie umgehend isoliert und mittels PCR getestet. Bei konsequenter Anamneseerhebung und Isolation sowie Testung aller symptomatischen Patienten beschränkt sich diese nachträgliche Testung unserer Erfahrung nach auf seltene Zufallsbefunde (z. B. Pneumonie in der CT-Traumaspirale oder Röntgen-Thorax bei Tumorsuche). Intubierte, komatöse oder intoxikierte Patienten vom Rettungsdienst werden prophylaktisch schutzisoliert und abgestrichen. Im Fall einer ohnehin notwendigen Schnittbildgebung wird diese großzügig um eine Thorax-CT erweitert. Bislang kam es unter diesem Vorgehen noch zu keiner signifikanten Eintragung von SARS-CoV‑2 auf die Bettenstationen. Natürlich wurde im Krisenstab ein generelles PCR-Screening aller Neuaufnahmen intensiv diskutiert. In Übereinstimmung mit den RKI-Empfehlungen erfolgt in unserem Klinikum jedoch weiterhin keine generelle Testung asymptomatischer Patienten aufgrund der Scheinsicherheit eines negativen Ergebnisses (geringe Sensitivität) und zur Schonung der Testkapazitäten.

## Patientenlenkung im Haus: Entlastung der Notaufnahme

Das UKM betreut aufgrund der Vielzahl an Spezialambulanzen und seiner hohen Bettenkapazität eine enorme Zahl an Patienten, die sich täglich durch das Klinikum bewegen. Insbesondere aus den Spezialambulanzen wurde eine Vielzahl von Fragen zum Umgang mit Reiserückkehrern aus Risikogebieten und zu immunsupprimierten Patienten mit respiratorischer Symptomatik an die Notaufnahme herangetragen. Zunächst mussten wir notgedrungen die Einbestellung überregionaler Spezialpatienten durch die jeweiligen Fachabteilungen sehr rasch unterbinden, sofern es sich nicht um einen medizinischen Notfall handelte, keine positive Risiko- oder Kontaktanamnese vorlag und das universitäre Leistungsspektrum nicht zwingend benötigt wurde. Für den Umgang mit elektiven Patienten wurde am 13.03.2020 eine dementsprechende SOP zur Patientenlenkung erarbeitet. Diese wurde, ergänzt durch den Screeningbogen der Notaufnahme, an sämtliche Pforten, Leitstellen, Ambulanzen und Pflegestützpunkte verteilt und die Mitarbeiter entsprechend geschult. Im konkreten Verdachtsfall wurde eine unmittelbare Vorstellung des Patienten beim Coronacontainer veranlasst. Durch diese Maßnahmen konnte der zusätzliche Zustrom von Patienten in die Notaufnahme effektiv reduziert werden. Nachdem im Verlauf der Ambulanzbetrieb auf reine Notfallpatienten beschränkt wurde, nahmen die hausinternen Anfragen an die Notaufnahme nochmals deutlich ab und beschränken sich aktuell auf wenige Spezialfälle bzw. -konstellationen.

## Diskussion

Trotz (oder gerade wegen?) vieler Informations-E-Mails zu einzelnen SOP oder Maßnahmen war die Durchdringung bei z. B. Hygienemaßnahmen und „physical distancing“ im Team lange Zeit nicht zufriedenstellend. Dieses Problem konnten wir erst durch zusätzliche Briefings, mehrere Teambesprechungen und „microteachings“ beseitigen. Rückblickend wäre eine frühere und noch persönlichere Kommunikation zwischen dem Leitungsteam und den verschiedenen Berufsgruppen in der Notaufnahme notwendig gewesen, um den unterschiedlichen Kenntnisstand bezüglich der COVID-19-Pandemie anzugleichen.

Obwohl wir frühzeitig in zahlreichen Fachabteilungen COVID-Ansprechpartner finden konnten, wurde die Bedeutung der Pandemie für die dortigen Abläufe (auch unabhängig von Notfällen) erst verzögert wahrgenommen. Zum Beispiel ist die Problematik der operativen Versorgung von Verdachts- bzw. COVID-Fällen erst mit 2 Wochen Verspätung aufgegriffen worden.

Obwohl wir alle im privaten Bereich schon sehr konkret an „physical distancing“ arbeiten, saßen wir in der Notaufnahme retrospektiv zu lange in zu kleinen Arztzimmern zusammen. Aktuell ziehen wir in zusätzliche Büros in einem Nachbarflur ein. Zusätzlich tragen seit Ende März alle (!) Mitarbeiter im UKM einen Mund-Nasen-Schutz.

Wir mussten feststellen, dass für die korrekte Verwendung der individuellen Schutzausrüstung erheblich mehr Schulungsbedarf besteht als geahnt. Hier versuchen wir gerade, eine neue Fehlerkultur einzuführen. Ein Ansatz ist die wechselseitige Kontrolle im Rahmen des An- und Ablegens der Schutzkleidung.

Derzeit optimieren wir unsere bestehenden Ausfallskonzepte, um Pflegende und Ärzte aus anderen Bereichen für den Einsatz in der Notaufnahme vorzubereiten. Als Lehrnotaufnahme einer großen Uniklinik bestand zudem die Möglichkeit, innerhalb von nur 2 Tagen die Zahl der PJ-Studenten in der Notaufnahme von 4 auf 8 zu verdoppeln. Es besteht eine enge Kommunikation mit dem Dekanat der medizinischen Fakultät zwecks Koordination weiterer studentischer Hilfskräfte im Bedarfsfall.

Etwas Gutes hat die COVID-Pandemie zumindest mit sich gebracht – die Notaufnahme erfährt seit nunmehr 4 Wochen eine grandiose und extrem unbürokratische Unterstützung durch praktisch alle Bereiche des Unternehmens. Egal ob neue PC oder Telefone, IT-Support, zusätzliche Dienstleistungen oder Nachschub von knappen Verbrauchsmaterialien – die Wege sind deutlich kürzer geworden und Probleme werden sehr zeitnah und pragmatisch gelöst (Übersicht siehe Tab. 1); eine sehr schöne Erfahrung, die durch die zahlreichen neuen Kontakte sicherlich weit über die Pandemie hinaus Bestand hat.

**Table 1:** 

*Strukturelle Maßnahmen*	Mitarbeit im Krisenstab
	Koordination Klinik/Hygiene/Virologie via CAMS
	Etablierung SARS-CoV-2-PCR
	Optimierung Probenversand
	Personalerhöhung Virologie (MTA aus Forschung)
	Isolationszimmer/Unterdruck/räumliche Separation
	Zusätzliche Isolationsstation
*Patientenorientierte Maßnahmen*	Container für Verdachtsfälle
	Studentische Telefonhotline
	Screeningfragebogen bei Aufnahme
	Quarantäneinfomaterial
	Flussschemata (Allokation und Testung)
	Vorgepackte Blutentnahmesets
	Sonderabsprache Funktionsbereiche
	Sicherstellung tel. Erreichbarkeit COVID-Beauftragte der Fachdisziplinen
	„Checkbox“ SARS-CoV-2-Infektiosität bei Anforderungen im KIS
*Mitarbeiterorientierte Maßnahmen*	Intensivierung der Teambesprechungen
	„Microteaching“
	Hygieneschulungen
	Regelmäßige Updates per E‑Mail
	Aufstockung und verstärkte Integration Medizinstudierende (PJ)
	Optimierung Ausfallkonzepte und Mitarbeiterschulung

*CAMS* Corona-Anamnese-MS(CAMS)-Formular, *MFA* Medizinische Fachangestellte, *SARS-CoV‑2* „severe acute respiratory syndrome coronavirus 2“, *PJ* Praktisches Jahr

## References

[1] https://corona.rki.de. Zugegriffen: 27.03.2020

[2] Kluge S, Janssens U, Welte T (2020). German recommendations for critically ill patients with COVID19. Med Klin Intensivmed Notfmed.

[3] Zhu N, Zhang D, Wang W (2020). A novel coronavirus from patients with pneumonia in China, 2019. N Engl J Med.

[4] Spina S, Marrazzo F, Migliari M (2020). The response of Milan’s emergency medical system to the COVID-19 outbreak in Italy. Lancet.

[5] Tuite AR, Ng V, Rees E (2020). Estimation of COVID-19 outbreak size in Italy. Lancet Infect Dis.

[6] Correa-Martinez CL, Kampmeier S, Kumpers P (2020). A pandemic in times of global tourism: superspreading and exportation of COVID-19 cases from a ski area in Austria. J Clin Microbiol.

[7] Zhou F, Yu T, Du R (2020). Lancet.

[8] Wu C, Chen X, Cai Y (2020). Risk factors associated with acute respiratory distress syndrome and death in patients with coronavirus disease 2019 pneumonia in Wuhan, China. JAMA Intern Med.

[9] Lippi G, Lavie CJ, Sanchis-Gomar F (2020). Cardiac troponin I in patients with coronavirus disease 2019 (COVID-19): Evidence from a meta-analysis. Prog Cardiovasc Dis.

